# Who is sceptical about emerging public health threats? Results from 39 national surveys in the United Kingdom

**DOI:** 10.1016/j.puhe.2015.09.004

**Published:** 2015-12

**Authors:** G.J. Rubin, Y. Finn, H.W.W. Potts, S. Michie

**Affiliations:** a King's College London, Department of Psychological Medicine, Weston Education Centre, Cutcombe Road, London SE5 9RJ, UK; b University College London, Centre for Health Informatics and Multiprofessional Education, UCL Institute of Health Informatics, London, UK; c University College London, Division of Psychology and Language Sciences, London, UK

**Keywords:** Pandemic, Scepticism, Communication, Psychology, Influenza

## Abstract

**Objectives:**

Members of the public are often sceptical about warnings of an impending public health crisis. Breaking through this scepticism is important if we are to convince people to take urgent protective action. In this paper we explored correlates of perceiving that ‘too much fuss’ was being made about the 2009/10 influenza A H1N1v (‘swine flu’) pandemic.

**Study design:**

A secondary analysis of data from 39 nationally representative telephone surveys conducted in the UK during the pandemic.

**Methods:**

Each cross-sectional survey (combined *n* = 42,420) collected data over a three day period and asked participants to state whether they agreed or disagreed that ‘too much fuss is being made about the risk of swine flu.’

**Results:**

Overall, 55.1% of people agreed or strongly agreed with this sentiment. Perceiving that too much fuss was being made was associated with: being male, being white, being generally healthy, trusting most in a primary care physician to provide advice, not knowing someone who had contracted the illness, believing you know a lot about the outbreak, not wishing to receive additional information about the outbreak and possessing worse factual knowledge about the outbreak than other people.

**Conclusions:**

In future disease outbreaks merely providing factual information is unlikely to engage people who are sceptical about the need to take action. Instead, messages which challenge their perceived knowledge and which present case studies of people who have been affected may prove more effective, especially when delivered through trusted channels.

## Highlights

• Data from 39 UK telephone surveys conducted during the ‘swine flu’ pandemic were analysed (*n* = 42,420).
• Scepticism was assessed by asking whether participants agreed that ‘too much fuss is being made about the risk of swine flu.’
• Around half of respondents were sceptical about the risk of swine flu (55.1%).
• Scepticism was associated with being white, healthy and male, and having high subjective knowledge about the outbreak.
• Challenging perceived knowledge may improve communication with sceptical groups in future.

## Introduction

It is sometimes assumed that warning people about an impending public health crisis will cause panic.^1^ In practice, apathy or scepticism is more common. The initial stages of the 2009/10 influenza A H1N1v (‘swine flu’) pandemic were a case in point. Despite concerns that the impending pandemic could be severe, and in the face of extensive (and largely accurate^2^) media coverage, surveys conducted in the UK found that over two-thirds of people thought that the media were over-exaggerating the situation^3^ while around half the population agreed with a statement that ‘too much fuss’ was being made.^4^ High levels of scepticism have also been found for other forms of urgent public health warning^5^ and been linked to ‘warning fatigue’^6^, ^7^, ^8^ and to a perception that health-related media reporting is based more on scaremongering than on accurate journalism.^9^, ^10^, ^11^

This widespread scepticism poses a problem for public health officials and organisations who may need to convince a population to engage in precautionary behaviour. During the swine flu pandemic, believing that the situation had been exaggerated was associated with a reduced intention to be vaccinated^4^ and a reduced likelihood of carrying out recommended behaviours such as washing hands more regularly.^3^ In any future crisis, public health officials and organisations will need to ensure that their communications reach sceptical people and are able to influence their thinking, emotions and behaviour.

Effective delivery of messages would be helped by identifying the demographic subgroups that are most likely to be sceptical and by identifying which people or organisations they most trust to inform them about health threats. Designing messages to influence their thinking, knowledge and behaviour may be more challenging, particularly if people who are sceptical about a health risk believe that they already possess sufficient knowledge about it and therefore do not need to attend to any new messages.^12^

In this study, we performed a secondary analysis of a dataset derived from a series of national telephone surveys conducted in the UK during the 2009/10 pandemic that contained data on perceptions that ‘too much fuss’ was being made about the pandemic. We tested whether these perceptions were associated with: a) specific demographic characteristics either during the pandemic as a whole or during three specific stages within it (the start, the peak and the tail-end); b) levels of trust in particular people or organisations; and c) the amount of perceived and actual knowledge a participant had about the outbreak and their perceived information needs in relation to it.

## Methods

### The surveys

Thirty-nine telephone surveys were conducted during the course of the pandemic by the Ipsos MORI Social Research Institute on behalf of the English Department of Health. Each collected data over three days. Surveys were conducted approximately weekly between 1 May 2009 and 14 February 2010. As the pandemic progressed, survey questions were modified or removed and new questions added to meet the Department of Health's evolving priorities. Random digit dialling and proportional quota sampling were used to ensure that the sample for each survey was demographically representative of the UK population. Quotas were set to ensure that the number of participants within given groups for age, sex, geographical region and social grade (a classification system based on the occupation of the chief income earner of a household) were equivalent to the known distribution of the UK population based on the latest census statistics. To be eligible for a survey, respondents had to be 16 years or over and speak English. Each survey was introduced to participants as ‘a national survey on a variety of subjects.’ Other topics were asked about only after all influenza-related questions had been covered. Response rates for each survey, calculated as the number of completed interviews divided by the total number of people spoken to, were in the region of 9–10%. These rates are not unusual for surveys of this nature and are not necessarily associated with high response bias.^13^ Other findings from this series of surveys are reported elsewhere.^4^, ^14^

### ‘Too much fuss’

Participants were asked whether they agreed or disagreed that ‘too much fuss is being made about the risk of swine flu.’ Response options were ‘strongly agree,’ ‘tend to agree,’ ‘neither agree nor disagree,’ ‘tend to disagree,’ ‘strongly disagree’ and ‘don't know.’

### Demographic characteristics

Demographic data recorded for each participant included their age, sex, ethnicity and social grade (using the categorisation of ‘ABC1’ [broadly managerial or professional] vs ‘C2DE’ [broadly manual or casual workers or unemployed on state benefit]).^15^ Participants were asked ‘how is your health in general’ (‘very good,’ ‘good,’ ‘fair,’ ‘poor,’ ‘very poor’) and ‘do you have any long-standing illness, disability or infirmity.’ Participants in 22 surveys (19 June to 19 July 2009, and 18 September 2009 to14 February 2010) were asked ‘Do you personally work for the NHS [National Health Service] in any capacity.’ Participants in 12 surveys (23 October 2009 to 14 February 2010) were asked whether anyone in the following groups had contracted swine flu: themselves; their children; or friends, colleagues or other family members.

### Trust

Participants in the first five surveys (1–17 May 2009) were asked ‘Which one of these would you trust most to advise you during a swine flu pandemic’ and were offered a list of options including ‘my doctor/GP,’ ‘NHS Direct [a telephone health helpline],’ ‘the Department of Health,’ ‘my local hospital,’ and ‘the government.’

### Perceived and actual knowledge, and stated information needs

Data for information-related variables were also drawn from the first five surveys. Participants were asked ‘how satisfied or dissatisfied are you with the amount of information available to you on swine flu, from any source,’ with possible responses being ‘very satisfied,’ ‘fairly satisfied,’ ‘neither satisfied nor dissatisfied,’ ‘fairly dissatisfied’ and ‘very dissatisfied.’ Participants were also asked: ‘how much have you heard about swine flu?’ (‘a lot,’ ‘a moderate amount,’ ‘a little,’ ‘nothing at all’) and ‘how much do you think you know about swine flu?’ (‘a lot,’ ‘a moderate amount,’ ‘a little,’ ‘nothing at all’). Five questions assessed whether participants had received a government leaflet about swine flu that was being distributed to every household in the country at the time of the surveys and whether they had heard or seen one of four types of government advertisement about the outbreak. To assess factual knowledge, participants were given six statements and asked to say if they were true, false or if they did not know. These (with the correct answers in brackets) were: currently there is no vaccine to protect against swine flu (true); there are ways to help slow the spread of swine flu (true); if swine flu breaks out, it is likely that some people will have natural immunity to it (false); the ordinary flu vaccine will protect me from swine flu (false); it is possible to catch swine flu from eating pork (false); thousands of people worldwide have died from swine flu (false). Participants were also asked to state ‘what additional information you would like to receive.’ Answers to this open-ended question were coded by the Ipsos MORI interviewers into one of 12 categories (e.g. ‘details on symptoms,’ ‘advice on prevention’).

### Analyses

In keeping with previous studies in this area,^16^ we recoded responses to the ‘too much fuss’ question into two categories in order to simplify the analysis: agree or disagree. We excluded ‘neither agree nor disagree’ responses. For all questions, we counted responses of ‘don't know’, ‘unsure’ or ‘not applicable’ as missing data.

For ease of interpretation, we combined some response options for some predictor variables (see Table 1, Table 2, Table 3, Table 4, Table 5 for details). Binary logistic regressions were used to identify significant predictors of perceiving that too much fuss had been made. A first set of regression analyses assessed the role of demographic variables as predictors, including after adjustment for all other demographic variables. We examined these associations for the entire dataset as well as in three pandemic periods: in the early stages of the outbreak before the first UK death had occurred (five surveys from 1 May to 17 May 2009), at the height of the first wave of illness to occur in the UK (three surveys from 10 to 26 July 2009) and at the tail end of the outbreak (four surveys from 8 January to 14 February 2010).

A second set of regression models was used to test the role of the trust, knowledge and information variables as predictors of perceiving that too much fuss had been made, while adjusting for demographic characteristics. For the six true or false items, we assessed each item individually and also created a variable which reflected the number of correct answers (0–6) that a participant gave.

## Results

**Fig. 1:**
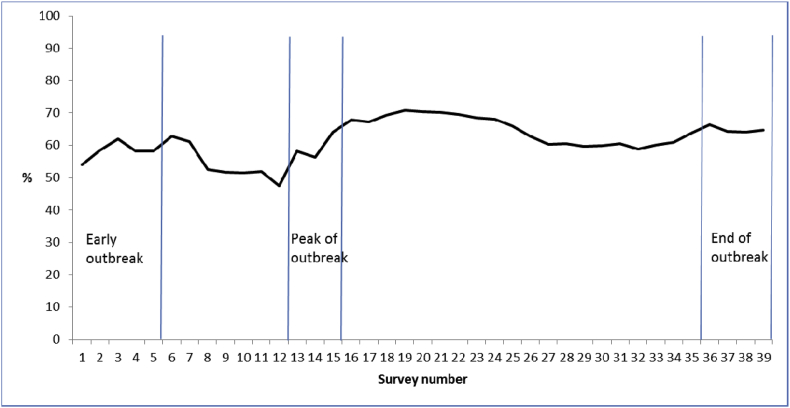
Percentage of respondents in each survey who strongly agreed or agreed that too much fuss was being made about the pandemic. Participants who neither agreed nor disagreed or who did not know were excluded.

Sample sizes for the surveys ranged from 1173 to 1047. The total sample size across all surveys was 42,420. Overall, 11,384 people (27.5%) strongly agreed that too much fuss was being made about the outbreak, 11,415 (27.6%) tended to agree, 3531 (8.5%) neither agreed nor disagreed, 8494 (20.5%) tended to disagree, 5729 (13.9%) strongly disagreed and 811 (2.0%) did not know. Fig. 1 shows the proportion over time who strongly agreed or agreed. This fluctuated between 47.4% and 70.9%.

### Association between demographic characteristics and too much fuss

The demographic characteristics of the sample and their associations with perceiving that too much fuss had been made are shown in Table 1. After adjusting for other demographic characteristics, people were more likely to perceive that too much fuss was being made if: they were male, they were aged 65 years or over, they were in a higher social grade, they were white, they had good general health and no chronic illness, and if they did not know a friend, colleague or family member who had contracted swine flu.

Table 2 shows these associations when analysed separately for the three pandemic periods. Broadly consistent associations over time were noted between believing that too much fuss had been made and being male or being 65 years or older. The association with social grade was not apparent at the tail end of the pandemic. The association with being white was only apparent at the start of the pandemic. Associations with general health were no longer apparent at any stage of the pandemic, while associations with the presence of a chronic illness were only apparent at the peak of the pandemic. Due to a lack of data, the association with not knowing a friend, colleague or family member who had been affected could only be assessed at the tail end of the pandemic, where a significant association was found. Although restricting the analyses to specific periods of time and hence reducing the sample size removed the statistical significance for several of the associations, the confidence intervals of the odds ratios for each time period overlapped with those for the other two time periods. This suggests that the pattern of associations remained reasonably consistent over the course of the pandemic.

### Association with trust

Table 3 shows the association with trust overall, participants reported that their most trusted sources for information about the outbreak were their doctor or general practitioner (2797 people, 51.6%), the NHS Direct telephone helpline (1137, 21.0%), the Department of Health (666, 12.3%) and their local hospital (238, 4.4%). Other responses, including the Government, the BBC, friends and family, and the media, were reported by fewer than 2% each. People who felt that too much fuss was being made were more likely to trust their family doctor and less likely to trust the Department of Health.

### Association with knowledge and information-related variables

Table 4 shows the associations with the knowledge and information-related variables. After adjusting for demographic characteristics, participants who believed that too much fuss was being made were more likely to say that they knew a lot about swine flu and that they had no additional information needs. They also answered fewer of the true or false questions correctly. Specific true or false answers that were associated with believing that too much fuss was being made were the beliefs that: if swine flu breaks out, some people will have natural immunity; the seasonal flu vaccine protects against swine flu; swine flu cannot be caught from pork; and that thousands of people had not died from swine flu. Receiving the government leaflet and encountering official advertising about the outbreak was not associated with believing that too much fuss had been made.

Table 5 shows the specific information needs reported by participants. Participants who felt that too much fuss was being made were less likely to request information for nearly every category (specifically: details on symptoms, advice on prevention, advice for people who need more tailored information, advice on treatment, what other countries are doing, availability of medicines or vaccines, how swine flu is spread, where to get access to a government leaflet and regular updates).

## Discussion

The belief that too much fuss was being made of the 2009/10 pandemic was common in the UK population from the start of the outbreak through to its conclusion. Although Fig. 1 suggests that some decreases in this sentiment were observed during periods that coincided with the summer and winter peaks of the outbreak, the overall stability of the belief was striking. The public appear to have started with an assumption that the danger associated with the pandemic was being over-stated, and little seems to have altered in that perception over the subsequent months. Neither official advertisements nor the government's door-to-door leaflet campaign had any impact on the perception of ‘too much fuss,’ highlighting the difficulty faced by public health officials when attempting to communicate to a sceptical public about an intangible, uncertain risk.

Our findings suggest some strategies that could be used to improve communication in future outbreaks. First, if our data generalise to other situations then it may possible to predict which sections of a population are most likely to be sceptical about a newly emerging health threat and hence target them for more or different forms of communication. As suggested by previous work, white men certainly fit into this category.^17^, ^18^ Those in a higher social grade were also more likely to feel that too much fuss was being made. At first glance, this seems to run contrary to assumptions that ‘healthism’ is rife in Britain's middle classes, with those in higher social grades being more pre-occupied by the importance of protecting their health.^19^ However, the fact that influenza is a ‘natural’ rather than ‘manmade’ risk may make it less concerning from this perspective, suggesting that normal ‘healthy’ behaviours such as eating well and exercising may be sufficient as protection, while the greater wealth, resources and access to health care available to people from a higher social grade may also render the prospect of catching flu appear less threatening. Participants in the oldest age group were also more likely to be sceptical. This was surprising given that older adults are at higher risk from flu and that other groups who are at higher risk, including those with poor general health and those with a chronic illness, were less likely to express scepticism. We can only speculate that recollections of more severe pandemics among our older participants may have contributed to the effect.

Although having had swine flu oneself did not have any effect on believing that too much fuss was being made, knowing someone else who had caught it did appear to reduce this belief. It is possible that this finding reflects a form of recall bias, with participants only remembering other people who have had swine flu if their symptoms seemed particularly severe. Nonetheless, the finding does suggest that using examples of people who have become ill may be one way of influencing a person's thoughts, emotions or behaviour despite any initial scepticism.

Our results showing that the family doctor was the most trusted source of advice about the outbreak correspond with previous research, which has found health care professionals to be highly trusted both in general^20^ and in the context of sudden health threats.^21^ Given that this was particularly true for people who felt that too much fuss was being made, future efforts to break through scepticism may work best if it is seen to be spearheaded or supported by primary care practitioners.

Participants who agreed that too much fuss was being made were more likely to believe that they knew a lot about swine flu and to say that they did not have any further information needs than people who were less sceptical. This is problematic. People who feel that they already have sufficient information to form a view about a health risk are less likely to engage with new information that they encounter.^12^, ^22^ In the 2009/10 pandemic, at least, this perceived information sufficiency was misplaced, with those who felt that too much fuss was being made also being more likely to give the wrong answers to the factual questions included in the survey. Given the low level of self-reported information needs among this group, merely presenting facts about a risk may be unlikely to engage them in any future public health crisis: demonstrating that they know less than they think they do may be a more effective way of motivating them to seek out additional information.

Identifying strategies to break through initial scepticism among the public is important if future warnings about impending public health crises are to be effective. While our analyses suggest several concepts that might be usefully explored as ways of tackling scepticism, developing these into usable communication messages will require additional work. Experimental work is now required to test the impact of messages which portray people who have experienced pandemic flu, motivate people to seek information, and are supported by trusted primary care figures.

### Limitations

Two caveats should be borne in mind when evaluating our results. First, the cross-sectional nature of our data means that we cannot infer causality in the associations that we identified. Second it is important to be circumspect about the generalisability of our data. Although they are a useful starting point for future research and for designing and targeting communication campaigns, whether our findings apply to influenza outbreaks only, or even to the 2009/10 outbreak only, is not known. Additional research may help to clarify both points.

### Conclusions

Our findings from the 2009/10 swine flu pandemic suggest that it is possible to predict which sections of a population are most likely to be sceptical and that highlighting common misperceptions and using case studies of people who have become ill may be useful techniques to use when engaging with this group in future infectious disease outbreaks.

## Author statements

### Acknowledgements

None.

### Ethical approval

Not required (secondary analysis of fully-anonymised data containing no sensitive information).

### Funding

This report is independent research commissioned and funded by the Department of Health Policy Research Programme ‘Improving Communication With the Public About Antivirals and Vaccination During the Next Pandemic’, 019/0060. The work also received support from the National Institute for Health Research Health Protection Research Unit (NIHR HPRU) in Emergency Preparedness and Response at King's College London in partnership with Public Health England (PHE). The views expressed are those of the author(s) and not necessarily those of the NHS, the NIHR, the Department of Health or Public Health England.

### Competing interests

None declared.

## Figures and Tables

**Table 1:** Demographic characteristics of sample, and the association between demographic characteristics and agreeing that too much fuss is being made about the risk of swine flu. Adjusted odds ratios are adjusted for gender, age, social grade, ethnicity, general health and presence of a chronic illness.

Variable	Levels	Number (%) of participants	Odds ratio (95% confidence interval) for association with ‘too much fuss’	Adjusted odds ratio (95% confidence interval) for association with ‘too much fuss’
Gender	Male	17,296 (41.8%)	1.20 (1.15–1.25, *P* < 0.001)	1.20 (1.15–1.25, *P* < 0.001)
Female	24,068 (58.2%)	Reference category	Reference category

Age in years	16 to 24	3695 (8.9%)	1.03 (0.95–1.11, *P* = 0.56)	0.94 (0.86–1.02, *P* = 0.15)
25 to 34	4751 (11.5%)	0.94 (0.87–1.01, *P* = 0.09)	0.86 (0.80–0.93, *P* < 0.001)
35 to 54	14,096 (34.1%)	0.88 (0.83–0.93, *P* < 0.001)	0.81 (0.77–0.86, *P* < 0.001)
55 to 64	7652 (27.0%)	0.95 (0.89–1.02, *P* = 0.13)	0.92 (0.86–0.98, *P* = 0.01)
65 or older	11,170 (27.0%)	Reference category	Reference category

Social grade	ABC1	23,217 (56.1%)	1.16 (1.11–1.21, *P* < 0.001)	1.14 (1.09–1.19, *P* < 0.001)
C2DE	18,147 (43.9%)	Reference category	Reference category

Ethnicity	White	38,266 (92.6%)	1.19 (1.10–1.28, *P* < 0.001)	1.20 (1.10–1.30, *P* < 0.001)
Other ethnicity	3050 (7.4%)	Reference category	Reference category

General health status	Good or very good	31,568 (76.6%)	1.37 (1.27–1.48, *P* < 0.001)	1.28 (1.18–1.40, *P* < 0.001)
Fair	6548 (15.9%)	1.18 (1.08–1.29, *P* < 0.001)	1.13 (1.03–1.24, *P* = 0.01)
Poor or very poor	3111 (7.5%)	Reference category	Reference category

Presence of a chronic illness	Yes	12,598 (30.6%)	0.86 (0.82–0.90, *P* < 0.001)	0.92 (0.88–0.98, *P* = 0.004)
No	28,594 (69.4%)	Reference category	Reference category

I have had swine flu	Yes	424 (3.3%)	0.86 (0.70–1.06, *P* = 0.148)	0.91 (0.73–1.12, *P* = 0.36)
No	12,286 (96.7%)	Reference category	Reference category

My children have had swine flu (analyses restricted to parents)	Yes	208 (7.4%)	0.97 (0.72–1.3, *P* = 0.85)	1.04 (0.76–1.41, *P* = 0.83)
No	2590 (92.6%)	Reference category	Reference category

Friends, colleagues or other family members have had swine flu	Yes	4140 (32.6%)	0.85 (0.78–0.92, *P* < 0.001)	0.85 (0.78–0.93, *P* < 0.001)
No	8570 (67.4%)	Reference category	Reference category

Do you work for NHS	Yes	1266 (5.4%)	0.88 (0.78–0.997, *P* = 0.045)	0.92 (0.81–1.04, *P* = 0.19)
No	22,003 (94.6%)	Reference category	Reference category

**Table 2:** Association between demographic characteristics and agreeing that too much fuss is being made about the risk of swine flu at three time points. Adjusted odds ratios are adjusted for gender, age, social grade, ethnicity, general health and presence of a chronic illness.

Variable	Levels	Adjusted odds ratio (95% confidence interval) for association with ‘too much fuss’ (1–17 May 2009)	Adjusted odds ratio (95% confidence interval) for association with ‘too much fuss’ (10–26 July 2009)	Adjusted odds ratio (95% confidence interval) for association with ‘too much fuss’ (8 Jan to 14 Feb 2010)
Gender	Male	1.27 (1.13–1.42, *P* < 0.001)	1.26 (1.08–1.47, *P* = 0.03)	1.15 (1.01–1.33, *P* = 0.04)
Female	Reference category	Reference category	Reference category

Age in years	16 to 24	0.75 (0.60–0.94, *P* = 0.01)	0.94 (0.69–1.27, *P* = 0.67)	0.81 (0.61–1.07, *P* = 0.14)
25 to 34	0.73 (0.59–0.89, *P* = 0.003)	0.85 (0.64–1.12, *P* = 0.24)	0.78 (0.60–1.002, *P* = 0.05)
35 to 54	0.82 (0.70–0.96, *P* = 0.01)	0.74 (0.60–0.90, *P* = 0.03)	0.77 (0.62–0.93, *P* = 0.007)
55 to 64	0.89 (0.75–1.07, *P* = 0.22)	0.98 (0.78–1.24, *P* = 0.89)	0.77 (0.62–0.94, *P* = 0.01)
65 or older	Reference category	Reference category	Reference category

Social grade	ABC1	1.17 (1.04–1.32, *P* = 0.01)	1.28 (1.09–1.50, *P* = 0.002)	1.01 (0.87–1.16, *P* = 0.93)
C2DE	Reference category	Reference category	Reference category

Ethnicity	White	1.42 (1.12–1.79, *P* = 0.003)	0.98 (0.72–1.34, *P* = 0.90)	1.02 (0.78–1.32, *P* = 0.91)
Other ethnicity	Reference category	Reference category	Reference category

General health status	Very good or good	1.19 (0.94–1.53, *P* = 0.15)	1.21 (0.90–1.64, *P* = 0.21)	1.01 (0.75–1.37, *P* = 0.94)
Fair	1.13 (0.88–1.46, *P* = 0.36)	1.20 (0.87–1.66, *P* = 0.26)	0.94 (0.69–1.29, *P* = 0.69)
Poor or very poor	Reference category	Reference category	Reference category

Presence of a chronic illness	Yes	0.89 (0.76–1.03, *P* = 0.89)	0.80 (0.65–0.97, *P* = 0.02)	0.85 (0.71–1.02, *P* = 0.08)
No	Reference category	Reference category	Reference category

I have had swine flu	Yes	Question not asked	Question not asked	0.85 (0.60–1.22, *P* = 0.39)
No	Question not asked	Question not asked	Reference category

My children have had swine flu (analyses restricted to parents)	Yes	Question not asked	Question not asked	1.40 (0.78–2.51, *P* = 0.25)
No	Question not asked	Question not asked	Reference category

Friends, colleagues or other family members have had swine flu	Yes	Question not asked	Question not asked	0.75 (0.65–0.88, *P* < 0.001)
No	Question not asked	Question not asked	Reference category

Do you work for NHS	Yes	Question not asked	0.78 (0.51–1.21, *P* = 0.27)	1.04 (0.78–1.39, *P* = 0.79)
No	Question not asked	Reference category	Reference category

**Table 3:** Most trusted sources of information about swine flu, exposure to official information sources and their association with agreeing that too much fuss is being made about the risk of swine flu. Adjusted odds ratios were adjusted for age, sex, ethnicity, social grade, general health status and presence of a chronic illness.

Variable	Levels	Number (%) of participants	Odds ratio (95% confidence interval) for association with believing that too much fuss had been made	Adjusted odds ratio (95% confidence interval) for association with believing that too much fuss had been made
I would trust my doctor/general practitioner most to advise me during a swine flu pandemic	Yes	2622 (48.4%)	1.18 (1.05–1.32, *P* = 0.005)	1.16 (1.03–1.30, *P* = 0.02)
No	2797 (51.6%)	Reference category	Reference category
I would trust the NHS Direct telephone helpline most to advise me during a swine flu pandemic	Yes	1137 (21.0%)	0.89 (0.77–1.02, *P* = 0.09)	0.89 (0.77–1.02, *P* = 0.09)
No	4282 (79.0%)	Reference category	Reference category
I would trust the Department of health most to advise me during a swine flu pandemic	Yes	666 (12.3%)	0.79 (0.67–0.94, *P* = 0.008)	0.82 (0.68–0.97, *P* = 0.02)
No	4753 (87.7%)	Reference category	Reference category
I would trust my local hospital most to advise me during a swine flu pandemic	Yes	238 (4.4%)	0.91 (0.69–1.19, *P* = 0.48)	0.99 (0.75–1.31, *P* = 0.92)
No	5181 (95.6%)	Reference category	Reference category

**Table 4:** Knowledge about the pandemic and its association with agreeing that too much fuss is being made about the risk of swine flu. Adjusted odds ratios were adjusted for age, sex, ethnicity, social grade, general health status and presence of a chronic illness.

Variable	Levels	Number (%) of participants	Odds ratio (95% confidence interval) for association with believing that too much fuss had been made	Adjusted odds ratio (95% confidence interval) for association with believing that too much fuss had been made
Satisfaction with amount of information available	Very or fairly satisfied	4462 (91.0%)	1.07 (0.88–1.31, *P* = 0.50)	1.00 (0.81–1.23, *P* = 0.99)
Very or fairly dissatisfied	441 (9.0%)	Reference category	Reference category

How much have you heard about swine flu	A lot	3399 (65.6%)	1.27 (1.01–1.60, *P* = 0.04)	1.21 (0.95–1.53, *P* = 0.12)
A moderate amount	1418 (27.4%)	1.07 (0.84–1.37, *P* = 0.57)	1.04 (0.81–1.34, *P* = 0.76)
A little or nothing	366 (7.1%)	Reference category	Reference category

How much do you think you know about swine flu	A lot	1167 (22.6%)	1.50 (1.27–1.78, *P* < 0.001)	1.48 (1.24–1.76, *P* < 0.001)
A moderate amount	2636 (51.0%)	1.14 (0.99–1.30, *P* = 0.07)	1.11 (0.96–1.28, *P* = 0.17)
A little or nothing	1365 (26.4%)	Reference category	Reference category

Have you received the swine flu leaflet?	Yes	2004 (37.8%)	1.01 (0.90–1.14, *P* = 0.82)	0.99 (0.88–1.12, *P* = 0.89)
No	3291 (62.2%)	Reference category	Reference category

Have you seen official adverts about swine flu?	Number of adverts seen (0–4)^a^	Median: 1.0 (0–4)	1.02 (0.96–1.09, *P* = 0.45)	1.03 (0.97–1.09, *P* = 0.38)

Overall knowledge about swine flu	Sum of correct true/false answers^a^	Median: 5 (range 0–6)	0.92 (0.86–0.99, *P* = 0.02)	0.90 (0.84–0.97, *P* = 0.004)

There is currently no vaccine for swine flu	True	2597 (52.4%)	0.96 (0.86–1.08, *P* = 0.53)	0.97 (0.86–1.09, *P* = 0.61)
False	2360 (47.6%)	Reference category	Reference category

There are ways to slow the spread of swine flu	True	4878 (94.3%)	0.98 (0.77–1.26, *P* = 0.89)	0.93 (0.72–1.21, *P* = 0.60)
False	293 (5.7%)	Reference category	Reference category

If swine flu breaks out some people will have natural immunity	True	2917 (62.9%)	1.69 (1.49–1.92, *P* < 0.001)	1.69 (1.49–1.92, *P* < 0.001)
False	1722 (37.1%)	Reference category	Reference category

Ordinary flu vaccine will protect me	True	765 (14.1%)	1.31 (1.11–1.55, *P* = 0.001)	1.36 (1.14–1.61, *P* < 0.001)
False	4072 (84.2%)	Reference category	Reference category

Swine flu can be caught from pork	True	271 (5.2%)	0.56 (0.44–0.73, *P* < 0.001)	0.63 (0.48–0.83, *P* = 0.001)
False	4898 (94.8%)	Reference category	Reference category

Thousand have died from swine flu	True	529 (10.0%)	0.63 (0.52–0.76, *P* < 0.001)	0.67 (0.55–0.81, *P* < 0.001)
False	4749 (90.0%)	Reference category	Reference category

a Variable entered into regression model as continuous data.

**Table 5:** Information needs about the pandemic and their association with agreeing that too much fuss is being made. Adjusted odds ratios were adjusted for age, sex, ethnicity, social grade, general health status and presence of a chronic illness.

Information need	Levels	Number (%) of participants	Odds ratio (95% confidence interval) for association with believing that too much fuss had been made	Adjusted odds ratio (95% confidence interval) for association with believing that too much fuss had been made
Details on symptoms	Requested	594 (11.0%)	0.53 (0.44–0.63, *P* < 0.001)	0.56 (0.47–0.68, *P* < 0.001)
Not requested	4825 (89.0%)	Reference category	Reference category

Advice on prevention	Requested	440 (8.1%)	0.58 (0.48–0.71, *P* < 0.001)	0.63 (0.51–0.77, *P* < 0.001)
Not requested	4979 (91.9%)	Reference category	Reference category

Advice for people who might need more tailored information, e.g. those with pre-existing conditions	Requested	112 (2.1%)	0.54 (0.36–0.82, *P* = 0.003)	0.57 (0.38–0.86, *P* = 0.008)
Not requested	5307 (12.8%)	Reference category	Reference category

Advice on treatment	Requested	417 (7.7%)	0.58 (0.47–0.71, *P* < 0.001)	0.62 (0.50–0.77, *P* < 0.001)
Not requested	5002 (12.1%)	Reference category	Reference category

Travel advice	Requested	58 (1.1%)	0.58 (0.34–0.98, *P* = 0.04)	0.60 (0.35–1.03, *P* = 0.06)
Not requested	5361 (98.9%)	Reference category	Reference category

What other countries are doing	Requested	34 (0.6%)	0.25 (0.11–0.56, *P* = 0.001)	0.27 (0.12–0.60, *P* = 0.001)
Not requested	5385 (99.4%)	Reference category	Reference category

Availability of medicine or vaccine	Requested	69 (1.3%)	0.57 (0.34–0.95, *P* = 0.03)	0.54 (0.32–0.91, *P* = 0.02)
Not requested	5350 (98.7%)	Reference category	Reference category

How is swine flu spread	Requested	50 (0.9%)	0.50 (0.27–0.91, *P* = 0.02)	0.54 (0.29–0.995, *P* = 0.048)
Not requested	5369 (99.1%)	Reference category	Reference category

How many people or places are affected	Requested	68 (1.3%)	0.72 (0.44–1.19, *P* = 0.20)	0.70 (0.42–1.16, *P* = 0.17)
Not requested	5350 (98.7%)	Reference category	Reference category

A leaflet [circulated to all households during this period]	Requested	212 (3.9%)	0.65 (0.48–0.86, *P* = 0.003)	0.68 (0.51–0.91, *P* = 0.009)
Not requested	5207 (96.1%)	Reference category	Reference category

Information on outbreaks in the local area	Requested	124 (2.3%)	1.02 (0.69–1.50, *P* = 0.94)	0.98 (0.66–1.45, *P* = 0.91)
Not requested	5295 (97.7%)	Reference category	Reference category

Regular updates	Requested	156 (2.9%)	0.68 (0.49–0.96, *P* = 0.03)	0.70 (0.49–0.98, *P* = 0.04)
Not requested	5263 (97.1%)	Reference category	Reference category

I have no additional information needs	Has no information needs	3059 (56.4%)	1.92 (1.71–2.15, *P* < 0.001)	1.85 (1.64–2.08, *P* < 0.001)
Has information needs	2360 (43.6%)	Reference category	Reference category
